# Snowed In: A Case Report on the Utilization of Ultrasound in the Diagnosis of Fractures

**DOI:** 10.7759/cureus.33758

**Published:** 2023-01-13

**Authors:** Keith Hansen, Trevine Albert, Jonathan Quinonez, Samir Ruxmohan

**Affiliations:** 1 Osteopathic Neuromusculoskeletal Medicine, Larkin Community Hospital Palm Springs Campus, Hialeah, USA; 2 Interventional Pain, Larkin Community Hospital, Miami, USA; 3 Neurology/Osteopathic Neuromusculoskeletal Medicine, Larkin Community Hospital, Miami, USA; 4 Division of Neurocritical Care, UT Southwestern Medical Center, Dallas, USA; 5 Neurology, Larkin Community Hospital, Miami, USA

**Keywords:** general radiology, both-bone forearm fracture, omm research, ultrasound anatomy, plain radiography

## Abstract

The standard convention for diagnosing bone fractures is through radiography. However, radiography can miss fractures depending on the type of injury or if human error is present. This may be due to improper patient positioning leading to superimposing bones being captured in the image, obscuring pathology. As of late, ultrasound has been gaining traction in terms of its utilization for diagnosing fractures, which radiography can miss at times. Here we present a case of a 59-year-old female who was diagnosed using ultrasound with an acute fracture that was initially missed on X-ray.

We present a case of a 59-year-old female with a past medical history significant for osteoporosis who presented to an outpatient clinic for evaluation of acute left forearm pain. She reported sustaining a mechanical fall forward to the ground three weeks before bracing herself with her forearms, immediately developing left upper extremity pain lateralized to the forearm. Upon initial evaluation, forearm radiographs were obtained and showed no evidence of acute fractures. She then underwent a diagnostic ultrasound that showed an obvious fracture of the proximal radius, distal to the radial head. Upon reviewing initial radiograph films, it was evident that the proximal ulna was superimposed over the radius fracture as a proper neutral anteroposterior view of the forearm was not taken. The patient then underwent a computed tomography (CT) scan of her left upper extremity, which confirmed the presence of a healing fracture.

We present a case in which ultrasound is an excellent adjunct when a fracture cannot be identified on plain film radiography. Its utilization should be well-known and considered more often in the outpatient setting.

## Introduction

Early fracture diagnosis can help identify and mitigate sources of pain within trauma patients. Typically, the standard of management in the outpatient setting is multiple-view radiography depending on the type of injury present. For example, identifying an elbow fracture will require both anterior-posterior and lateral radiographs. This method, sadly, has its drawbacks due to radiation exposure and a physician’s ability to interpret radiographs. This method may be costly concerning time and clinical management.

In contrast, ultrasonography is now an alternative method for diagnosing bone fractures [1]. Since the 1980s, ultrasound has been utilized in the emergency department setting to assess chest wall trauma but has expanded to assess pediatric and pregnancy-related traumas via the focused assessment with sonography in trauma (FAST) examination [2]. Ultrasonography has also been endorsed by the World Health Organization as an alternative method for diagnosing fractures in remote settings [3]. This is now possible due to improvements in ultrasound resolution and related technologies. Despite such improvements, ultrasound is not readily involved in the outpatient setting as it requires the skill of a well-versed operator to identify vital organs and related structures quickly [3]. A benefit of ultrasonography, however, is faster diagnosing of bone fractures. Here, we present a case report of ultrasonography identifying a left forearm fracture of a 59-year-old female in place of radiography.

## Case presentation

**Figure 1:**
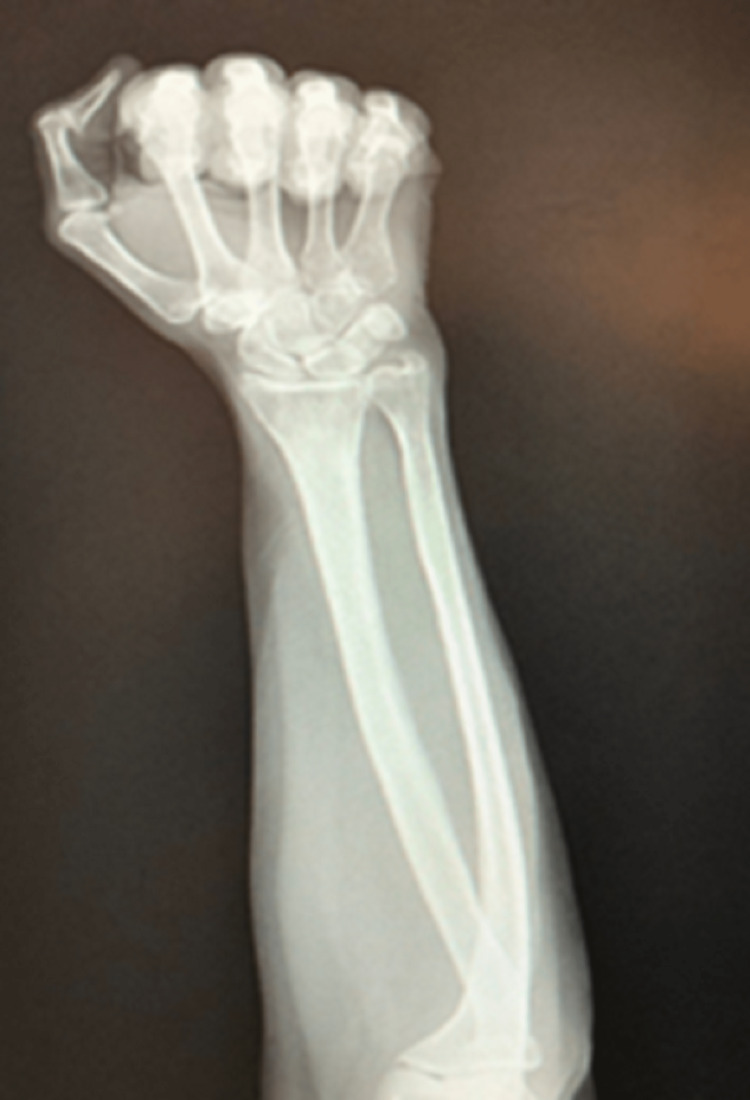
Left forearm radiograph - anterior-posterior view Radiograph of the patient’s left elbow and wrist from an anterior-posterior (AP) view. This radiograph does not show any immediate structural defects of the patient’s left radius, ulnar, carpal, and phalangeal bones. Other findings which are present include a small calcification within the left triangular fibrocartilage complex area near the left ulnar styloid process as well as a possible bone contusion about the left lateral epicondyle.

**Figure 2:**
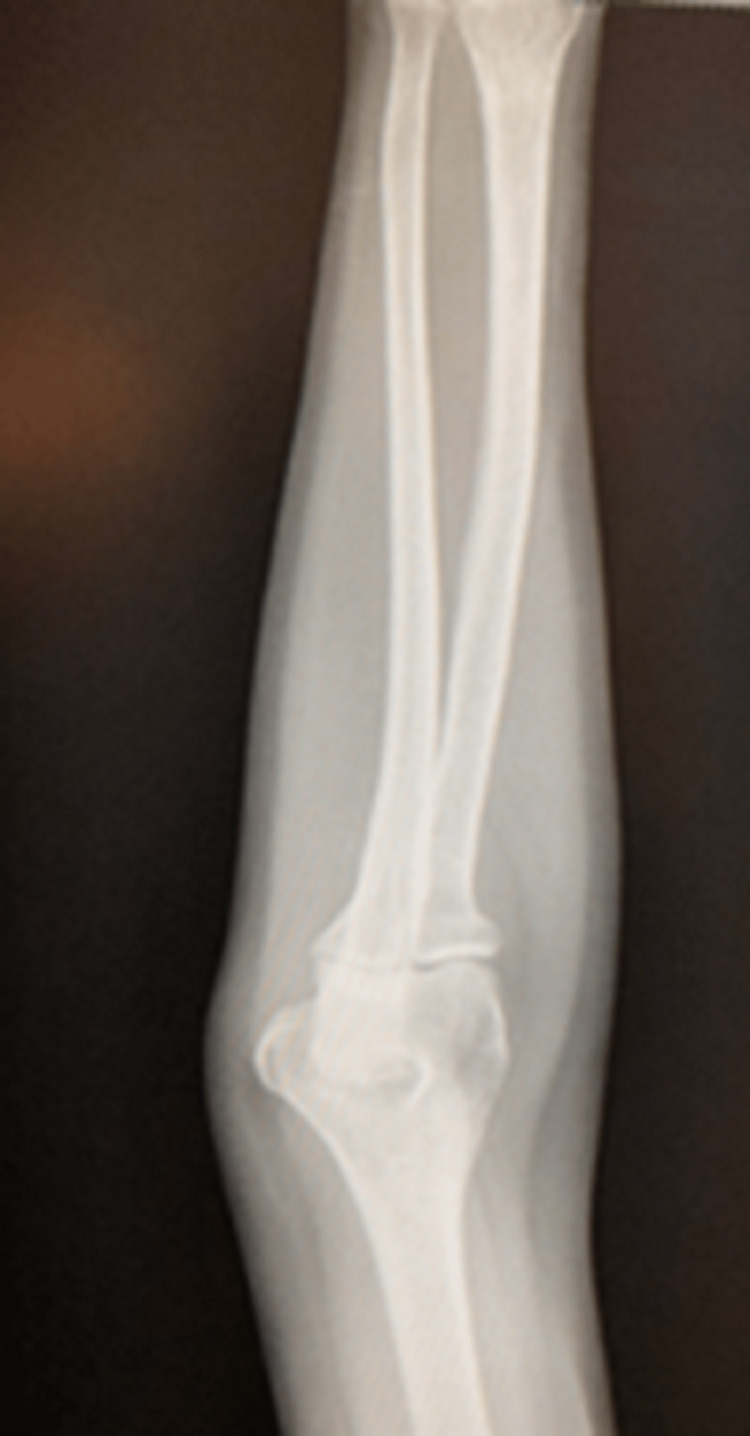
Left elbow radiograph - anterior-posterior view Radiograph of the patient’s left elbow from an anterior-posterior (AP) view. This radiograph does not show any immediate structural defects of the patient’s left radius, ulnar, or distal humerus bones.

**Figure 3:**
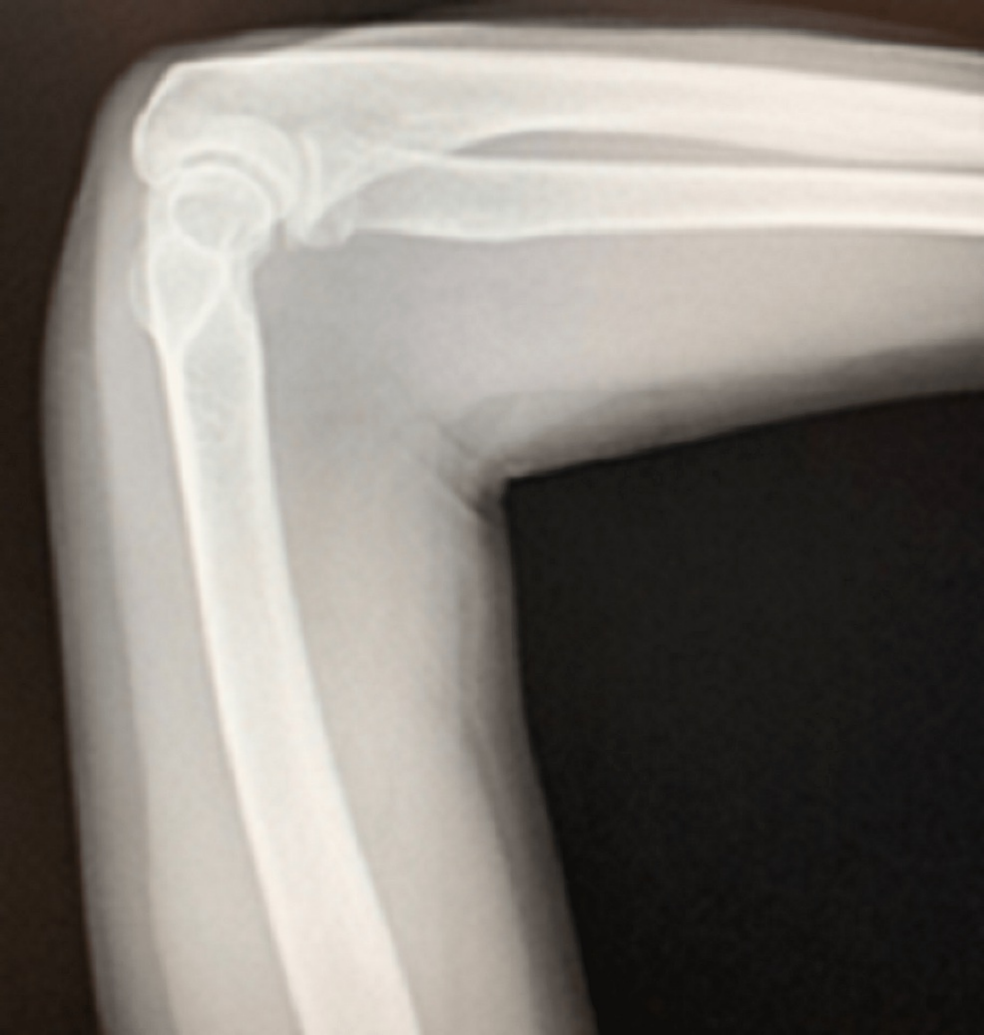
Left elbow radiograph - lateral view Radiograph of the patient’s left elbow in a lateral view. This radiograph does not show any immediate structural defects of the patient’s left radius, ulnar, carpal, and distal humerus bones. Other findings which are present include a possible bone contusion about the left lateral epicondyle.

**Figure 4:**
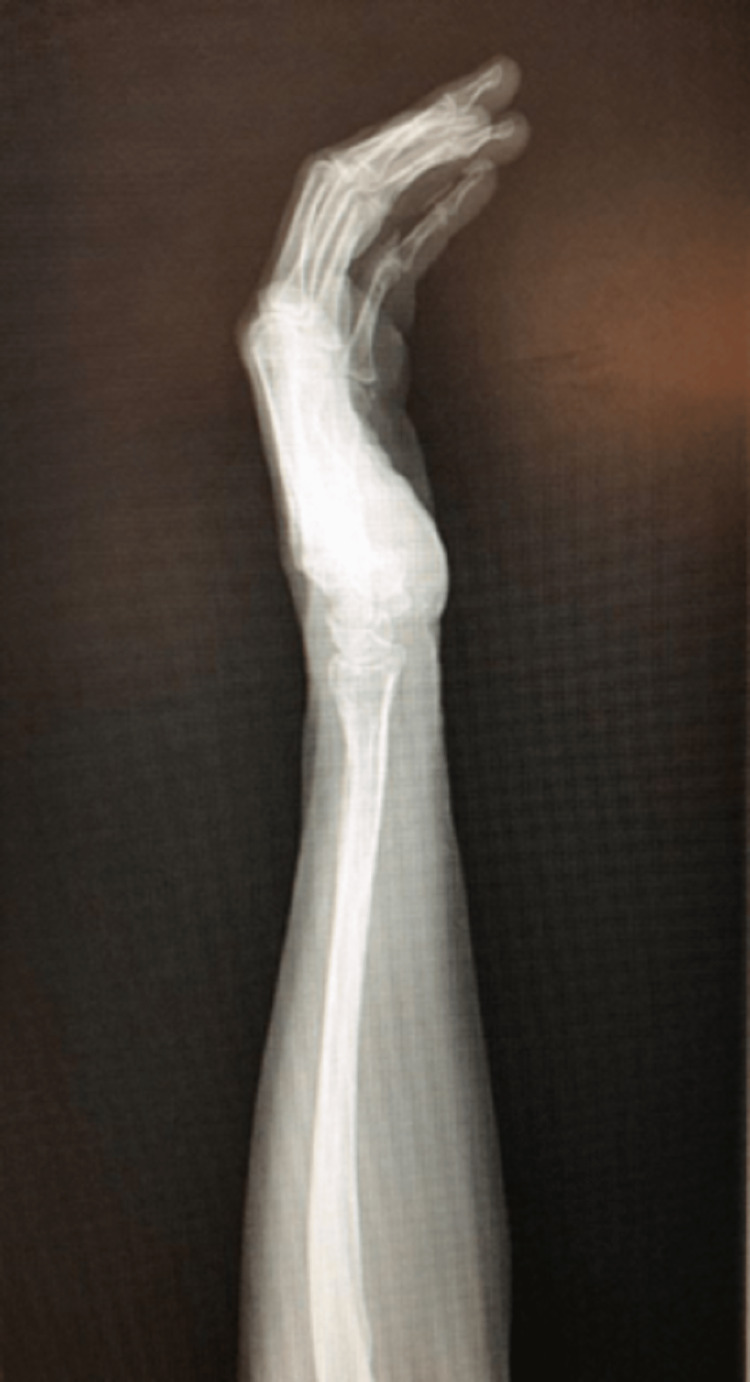
Left forearm and wrist radiograph - lateral view Radiograph of the patient’s left distal forearm and wrist in a lateral view. This radiograph does not show any immediate structural defects of the patient’s left radius, ulnar, carpal, and phalangeal bones.

We present a case of a 59-year-old female with a past medical history of osteoporosis who presented to an outpatient clinic for evaluation of acute left upper extremity pain for two weeks duration. Per the patient, she fell spontaneously and landed on her bilateral forearms and elbows. She immediately developed sudden left upper extremity pain involving her proximal shoulder to her distal wrist. The patient did not notice any edema or ecchymosis but endorsed an “aching pain” with both arm pronation and supination. She denied any numbness or tingling in her left upper extremity. She initially managed this pain with rest and ice. Upon presentation, her initial vital signs were within normal limits. A physical examination was unremarkable except for reproducible tenderness upon light and deep palpation throughout the left upper extremity. Reproducible tenderness was worse halfway along the left forearm. Left upper extremity radiography revealed the presence of no fractures in the left wrist or elbow. Radiography, however, demonstrated the presence of the left triangular fibrocartilage complex near the ulnar styloid process as well as a possible bone contusion about the left lateral epicondyle. Figures 1-4 show the presence of the above findings in multiple views.

**Figure 5:**
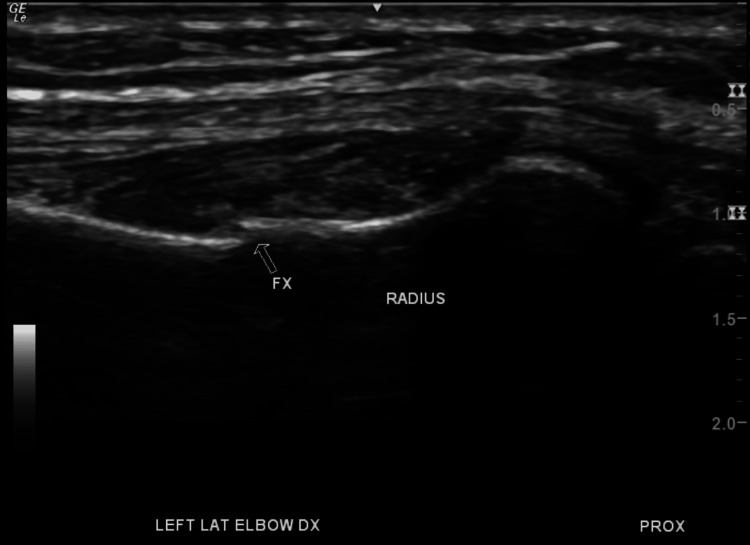
Forearm ultrasound with arrow showing the location of the fracture Ultrasound image of the patient’s left forearm. The figure shows a 1.5 mm cortical offset, indicating a possible fracture along the radial shaft distal to the radial head.

**Figure 6:**
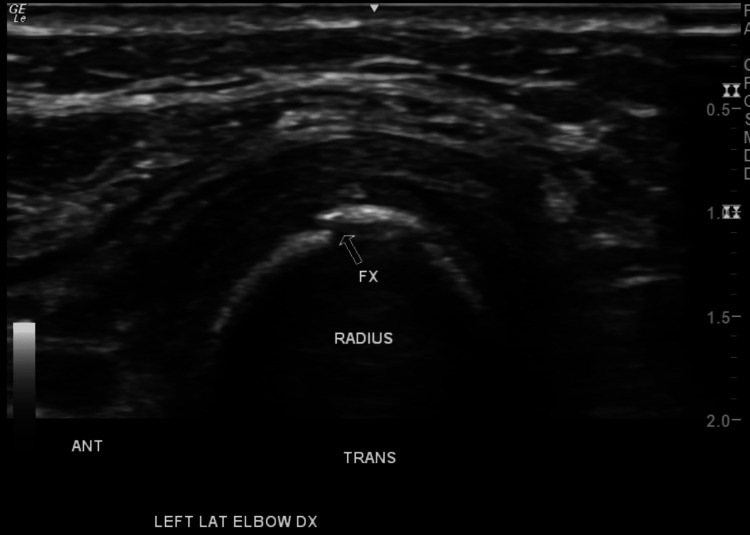
Forearm ultrasound with arrow showing the location of the fracture in a transverse view Ultrasound image of the patient’s left forearm in a transverse view. The figure shows a 1.5 mm cortical offset, indicating a possible fracture along the radial shaft distal to the radial head from a transverse view.

**Figure 7:**
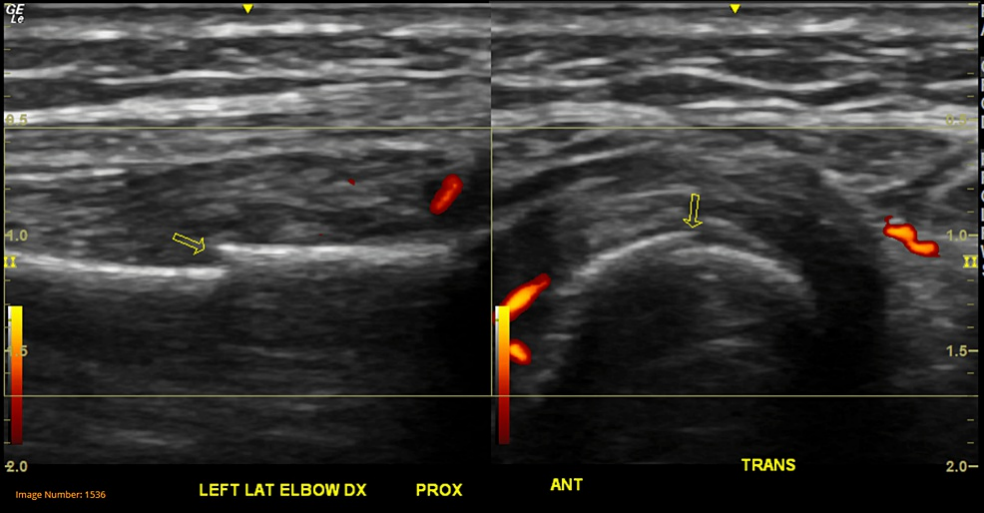
Forearm ultrasound showing blood flow near the site of the fracture Ultrasound image of the patient’s left forearm in longitudinal and transverse views. The figure shows arterial blood flow through the region with the fracture identified by the arrow. Doppler flow (shown in red) depicts no damage to surrounding structures.

Due to persistent pain, a left upper extremity musculoskeletal ultrasound was indicated, which revealed the following the presence of mild-moderate radiocarpal joint synovitis, extensor digitorum tendon tenosynovitis (wrist), and a 1-1.5 mm cortical bone offset along the radial shaft 1.5 cm distal to the radial head indicative of a possible fracture. Figures 5-7 show the presence of the radial fracture as identified by the arrow with Figure 7 showing the presence of intact vasculature; all images were produced using a 213 Hz General Electric ultrasound probe.

The patient later underwent computed tomography (CT) scan of the left upper extremity without contrast, which revealed a transversely oriented fracture through the neck of the proximal radius with 70% fracture healing. The patient was advised to follow up within 1-2 weeks for further management.

## Discussion

Prompt diagnosis of fractures can allow for accurate and efficient management. As seen in the case presented, ultrasonography allowed for an accurate diagnosis of the patient’s fracture and subsequent successful management. In acute traumatic situations leading to fractures, clinicians can gain from recognizing the importance of using ultrasonography as a better diagnostic tool versus radiography. A meta-analysis study performed by Yousefifard et al. showed that the sensitivity of ultrasonography in detecting fractures post-traumatic injury was greater than radiography (97% vs. 77%). The study results demonstrated that ultrasonography has higher diagnostic sensitivity with rib fractures than radiography [1]. This is due to the visualization of cortical bone deformities [1]. Researchers have investigated the value of using ultrasound in diagnosing upper extremity fractures. A study by Champagne et al. showed that the sensitivity of ultrasonography in the diagnosis of upper-extremity fractures is 93%, and the specificity is 93% [3-7]. Fractures are frequently encountered in emergency rooms and in the outpatient setting. Statistics show that among the United States population, the age-adjusted incidence of forearm fractures including wrist was 251.7/100 000 in women and 80/100 000 in men [3-7]. A trend showed an inclination towards older women. Moreover, the incidence of forearm fractures among the pediatric age group was about 10 per 1000 children [3-7]. Zhao et al. reported the excellent detection and diagnostic ability of ultrasound in the case of forearm, wrist, and hand fractures [3-7].

Recently, clinicians have considered ultrasonography to be a first-line diagnostic tool in response to emergent and traumatic scenarios [3-7]. While radiography is more commonly used in imaging and diagnosing bony injuries, unfortunately, radiography has a poorer diagnostic accuracy for certain types of fractures (e.g., scaphoid fractures) [3-7]. Furthermore, the main advantage of using ultrasonography in diagnosing fractures is eliminating radiation exposure, especially for children [3-7]. Another meta-analysis done by Schmid et al. showed that the accuracy of using ultrasonography in the diagnosis of fractures post-trauma was 91% sensitive and 94% specific [3-7]. Forearm fractures can be diagnosed with very high accuracy using ultrasonography versus fractures of the hands and feet, which necessitates further imaging as ultrasounds can often miss fractures in those areas [3-7].

## Conclusions

Ultrasound can be useful in diagnosing extremities fractures that are occult and missed on radiographs. Clinicians should consider ultrasonography in diagnosing fractures as it is more readily available and does not expose the patient to harmful radiation.
